# Spine Stereotactic Body Radiation Therapy Residual Setup Errors and Intra-Fraction Motion Using the Stereotactic X-Ray Image Guidance Verification System

**DOI:** 10.4236/ijmpcero.2014.31001

**Published:** 2014-02

**Authors:** Kosj Yamoah, Nicholas G. Zaorsky, Joshua Siglin, Wenyin Shi, Maria Werner-Wasik, David W. Andrews, Adam P. Dicker, Voichita Bar-Ad, Haisong Liu

**Affiliations:** 1 Department of Radiation Oncology, Jefferson Medical College and Kimmel Cancer Center of Thomas Jefferson University, Philadelphia, USA; 2 Department of Radiation Oncology, Fox Chase Cancer Center, Philadelphia, USA; 3 Department of Neurological Surgery, Jefferson Medical College, Philadelphia, USA

**Keywords:** Spine Stereotactic Body Radiotherapy, Immobilization, Intrafraction Motion

## Abstract

**Purpose:**

To determine the precision of our institution’s current immobilization devices for spine SBRT, ultimately leading to recommendations for appropriate planning margins.

**Methods:**

We identified 12 patients (25 treatments) with spinal metastasis treated with spine Stereotactic Body Radiation Therapy (SBRT). The Body-FIX system was used as immobilization device for thoracic (T) and lumbar (L) spine lesions. The head and shoulder mask system was used as immobilization device for cervical (C) spine lesions. Initial patient setup used the infrared positioning system with body markers. Stereotactic X-ray imaging was then performed and correction was made if the initial setup error exceeded predetermined institutional tolerances, 1.5 mm for translation and 2° for rotation. Three additional sets of verification X-rays were obtained pre-, mid-, and post-treatment for all treatments.

**Results:**

Intrafraction motion regardless of immobilization technique was found to be 1.28 ± 0.57 mm. The mean and standard deviation of the variances along each direction were as follows: Superior-inferior, 0.56 ± 0.39 mm and 0.77 ± 0.52 mm, (*p* = 0.25); Anterior-posterior, 0.57 ± 0.43 mm and 1.14 ± 0.61 mm, (*p* = 0.01); Left-right, 0.48 ± 0.34 mm and 0.74 ± 0.40 mm, (*p* = 0.09) respectively. There was a significantly greater difference in the average 3D variance of the BodyFIX as compared to the head and shoulder mask immobilization system, 1.04 ± 0.46 mm and 1.71 ± 0.52 mm; (*p* = 0.003) respectively.

**Conclusions:**

Overall, our institution’s image guidance system using stereotactic X-ray imaging verification provides acceptable localization accuracy as previously defined in the literature. We observed a greater intrafraction motion for the head and shoulder mask as compared with the BodyFIX immobilization system, which may be a result of greater C-spine mobility and/or the suboptimal mask immobilization. Thus, better immobilization techniques for C-spine SBRT are needed to reduce setup error and intrafraction motion. We are currently exploring alternative C-spine immobilization techniques to improve set up accuracy and decrease intrafraction motion during treatment.

## 1. Introduction

Spine metastasis occurs in up to 70% of all cancer patients, and approximately one-third may develop epidural extension or symptomatic cord compression [1]. Conventional external beam radiation therapy to the entire involved spine has been the standard of care of several decades. The spinal cord tolerance is often the dose-limiting factor to re-irradiation or dose escalation for the treatment of radioresistant lesions [2–4]. Spine stereotactic body radiation therapy (sSBRT) has become a viable therapeutic option for the delivery of a high dose of radiation to spine metastases while respecting the dose limits of the adjacent spinal cord. SBRT is defined as high dose per fraction radiation (>5 Gy per fraction) delivered to an image-guided target in 5 fractions or less using conformal radiation techniques [5]. Although very promising, the major challenge in the delivery of sSBRT is the close proximity of the dose-limiting spinal cord to the vertebral body, and spine metastases. Several preclinical studies have demonstrated the applicability of patient positioning, immobilization, and dosimetric characteristics of SBRT for spine metastases [6–8]. The feasibility of this approach was evaluated clinically which demonstrated targeting accuracy within 1.5 mm for actual patient treatment using various immobilization techniques [9–11].

In the supine treatment position, the thoracic (T)-spine and lumbar (L)-spine are most restricted in mobility due to the presence of the rigid ribcage and the patient’s abdominal weight respectively. However, the motion of cervical (C)-spine is relatively increased as it is restricted only by the head. While the vertebral column is easily visible on imaging and exhibits minimal physiologic organ movement, safe delivery of high dose of radiation requires not only precise targeting due to the proximity of the spinal cord, but also accurate treatment planning and delivery [5,12–14]. Therefore, accurate setup and immobilization as well as a reduction in intrafraction motion are required to minimize the spinal cord dose. The purpose of this study is to determine the precision of our institution’s current immobilization devices for spine SBRT, ultimately leading to recommendations for appropriate planning margins.

## 2. Methods

### 2.1. Patients

We identified 12 patients (25 treatments) with spinal metastasis treated with spine stereotactic body radiation therapy (SBRT). Patients were eligible for SBRT spine treatment if they had localized spine metastasis from the C1 to L5 levels on PET, CT, or MRI imaging. A minimum gap of ≥3 mm between the spinal cord and the edge of the epidural lesion was required. Patient with histology of myeloma or lymphoma, non-ambulatory based on spinal disease burden, rapid neurologic decline, spine instability due to a compression fracture, or spinal cord compression/displacement or epidural compression within 3 mm of the spinal cord were excluded. Dose range was from 15 – 25 Gy in 1 to 5 fractions. Total dose and fractionation regimen was determined at the treating physician’s discretion based on a number of factors including tumor location, proximity to spinal cord, normal structure tolerance and tumor histology.

### 2.2. Patient Immobilization and CT Simulation

The Elekta BodyFIX system was used as the immobilization device for T- and L-spine lesions. The Brain LAB head and shoulder mask system was used as the immobilization device for C-spine lesions. Five to six external infrared body markers for the BrainLAB ExacTRAC system were placed on the patient’s body (for BodyFIX immobilization) or on head/shoulder masks (for mask immobilization), as guidance for the initial setup using BrainLAB ExacTRAC infrared positioning system. All patients underwent a CT scan with the immobilization device and body markers. Four patients (12 treatments) with upper T-C spine lesions were treated using the BrainLAB head and shoulder mask immobilization system. A total of eight patients (13 treatments) with lower T-L spine lesions were treated using the BodyFIX immobilization system. All patients were treated in the supine position.

### 2.3. Treatment Planning

Target and critical structures were contoured according to Radiation Therapy Oncology Group (RTOG) 0631 guidelines. Image fusion between simulation CT and MRI (whenever possible) was used for delineation of both the soft tissue tumor component and the spinal cord. The conventional spinal cord was contoured with a superior border at least 10 cm above the superior extent of the target volume and an inferior boarder at least 10 cm below the inferior extent of the target volume, except in low lying L-spine lesions, in which case the caudaequina was drawn. 80% to 90% of the target volume received the prescribed dose. The dose constraints utilized for the spinal cord were 10 Gy to a spinal cord volume less than 0.35 cc and a maximum permitted cord point dose was 14 Gy (less than 0.03 cc). IMRT plans were generated using BrainLAB iPLAN (version 4.1) treatment planning system.

### 2.4. Treatment Delivery

Patients were treated with the Novalis stereotactic radio surgery system equipped with BrainLAB m^3^ micro-MLC on a Varian 600 C linear accelerator. BrainLAB ExacTRAC patient positioning system and robotic couch were used for patient setup. Initial patient setup used BrainLAB infrared positioning system with body markers. Stereotactic X-ray image guidance was then performed and correction was made if the initial setup error exceeded predetermined institutional tolerances, 1.5 mm for translation and 2° for rotation. Three additional sets of verification X-ray imaging were obtained pre, mid, and post treatment for all treatments. The calculated residual shifts and rotations were recorded to track intrafraction motion. The translational, rotational, and three-dimensional (3D) variances between pre-, mid-, and post- treatment imaging were calculated and used for data comparison.

### 2.5. Data Analysis

For each treatment, the maximum and minimum residual error along each orthogonal direction were extracted from three sets of data (pre-, mid-, and post-treatment verification images) and the difference between them was considered as the largest uncertainty in that direction. The root of sum of square (RSS) of the uncertainty along each of the three orthogonal directions was calculated and considered as the 3D uncertainty for that treatment. The mean and standard deviation of the uncertainty along each direction and in three dimensions were calculated and recorded. The difference of the mean uncertainty between C spine patients and T/L spine patients was were compared using an analysis of variance model.

## 3. Results

The disease characteristics are summarized in Table 1. Of the 12 patients analyzed in this study, 4 patients were treated to the C or T1 – T3 spine, 4 patients were treated to the T4 – T12 spine, and 4 patients were treated to the L1 – L5 spine. As shown in Table 2, the average and standard deviation in positioning error for translational degrees of freedom (Anterior-Posterior [AP], Superior-Inferior [SI], and Right-Left [RL]), and rotational degrees of freedom (pitch, roll, and yaw) are tabulated for upper T-C and lower T-L spine, respectively. There were 8 treatments that exceeded our tolerance of 1.5 mm error so that a manual correction was performed. Manual corrections were made solely based on the translational degrees of freedom since all of our patients in this study were within the acceptable rotational tolerance of 2°, as shown in Table 2. After the mid-treatment correction were made, the average residual error for these 8 treatments were 0.23 ± 0.2 mm (AP), 0.32 ± 0.2 mm (SI), 0.46 ± 0.3 mm (RL), and 0.66 ± 0.3 mm (3D) respectively. The average residual error post-treatment for these 8 treatments were 0.65 ± 0.6 mm (AP), 0.55 ± 0.5 mm (SI), 0.61 ± 0.5 mm (RL), and 1.22 ± 0.6 mm (3D) respectively. There was no significant difference in either translational or rotational degrees of freedom for lower T-L spine treatments.

**Figure 1:**
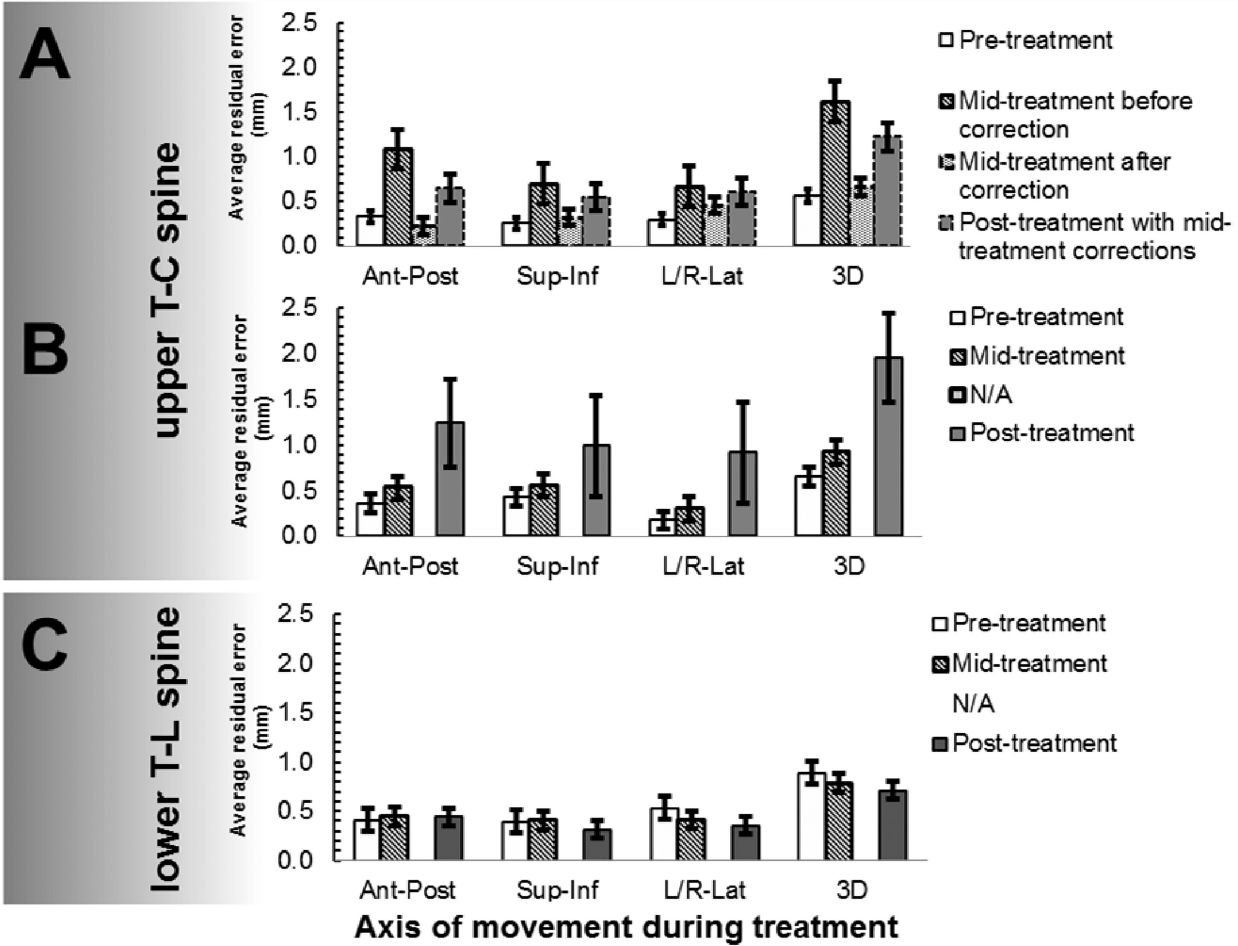
A comparison of pre-, mid-, post-treatment residual errors. Panels A and B show upper T-C spine treatments with mid-treatment-correction (n = 8), and without mid- treatment-correction (n = 4) respectively. Panel C shows lower T-L spine treatments without correction (n = 13).

Figures 1(A) and (B) display a comparison of the average pre-, mid-, post-treatment residual error for 8 treatments that required a mid-treatment correction to the 4 treatments that did not have a mid-treatment correction. There was no statistical difference between the residual error for pre-treatment vs. mid-treatment following correction (0.57 vs. 0.66 mm in 3D, *p* = 0.53). Furthermore, there was no statistical difference between the residual error for mid-treatments before-correction vs. post-treatment measurement (1.62 vs. 1.22 mm in 3D, *p* = 0.22), Figure 1(A). The average residual error post-treatment for the 4 treatments that required no correction after mid-treatment verification were 1.25 ± 0.5 mm (AP), 0.99 ± 0.6 mm (SI), 0.92 ± 0.6 mm (RL), and 1.96 ± 0.5 mm (3D) respectively, Figure 1(B). The average increase in residual error was most noticeable after the post-treatment verification (3D: 0.66 mm pre-treatment vs. 0.92 mm mid-treatment vs. 1.96 mm post-treatment). This suggests a large three-dimensional intra-fraction motion residual error from 0.66 mm pre-treatment to 1.96 mm post-treatment when no mid-treatment correction was applied.

Of the 8 patients, 13 treatments to the lower T-L spine, the average residual errors pre-treatment were 0.41 ± 0.3 mm (AP), 0.40 ± 0.3 mm (SI), 0.54 ± 0.4 mm (RL), and 0.89 ± 0.4 mm (3D) respectively. The average residual errors mid-treatment were 0.45 ± 0.3 mm (AP), 0.41 ± 0.3 mm (SI), 0.41 ± 0.3 mm (RL), and 0.79 ± 0.4 mm (3D) respectively. There were only 2 treatments that exceed our upper limit of acceptable residual error. The average residual errors post-treatment were 0.44 ± 0.2 mm (AP), 0.31 ± 0.3 mm (SI), 0.35 ± 0.3 mm (RL), and 0.71 ± 0.4 mm (3D) respectively (Figure 1(C); All *p* values > 0.3). The differences of the residual error between pre-, mid-, and post-treatment were not statistical significant in any direction, which shows that the lower T-L spine immobilization provides superior positional stability as compared to the upper T-C spine.

The average time span between the pre-treatment and mid-treatment verification for upper T-C spine is 5.8 ± 1.0 min, and the average time span between the mid- to post-treatment verification is 6.3 ± 1.7 min. There was no statistically significant difference between the pre- to mid- and mid- to post-treatment interval (*p* = 0.48). We observed that the average treatment time for upper T-C spine was significantly shorter than that for the lower T-L spine treatments. The average time span between the pre-treatment and mid-treatment verification for lower T-L spine is 9.5 ± 5.2 min and the average time span between the mid- to post-verification is 10.0 ± 4.4 min.

**Figure 2:**
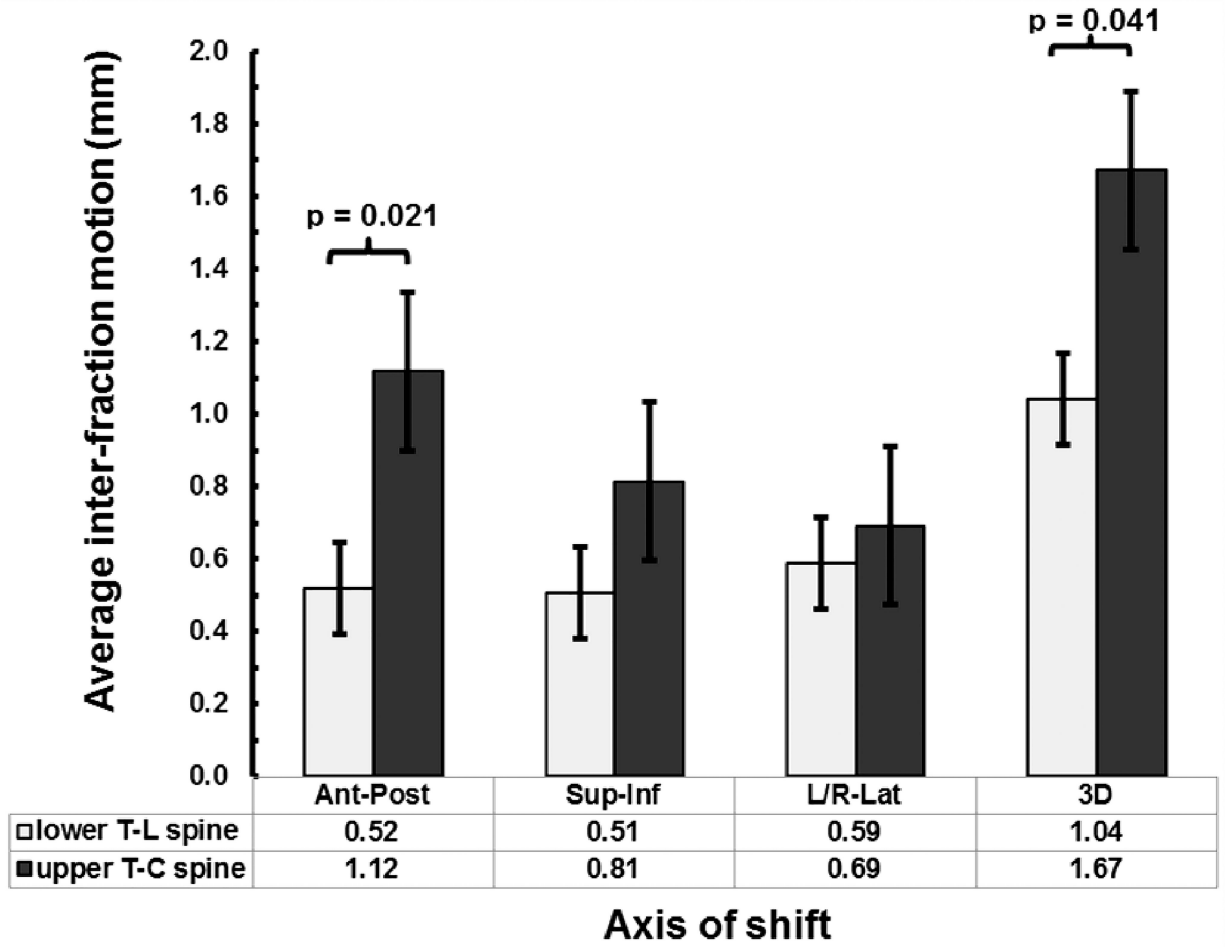
Comparison between the maximum intrafraction variation for upper T-C spine and lower T-L spine treatments along each direction and in 3D.

As shown in Table 3, intrafraction motion regardless of immobilization technique was 1.28 ± 0.57 mm. We compared upper T-C spine positional variation using BrainLAB mask immobilization system to lower T-L spine using Elekta BodyFIX immobilization system. Figure 2 represents the average maximum intrafraction variation along each direction and in 3D. We observed a significant difference in AP (0.52 mm vs. 1.12 mm, *p* = 0.02) and in 3D (1.04 mm vs. 1.67 mm, *p* = 0.04), but no significant difference in the SI (0.51 vs. 0.81, *p* = 0.2) and RL directions (0.59 vs. 0.69, *p* = 0.6).

**Figure 3:**
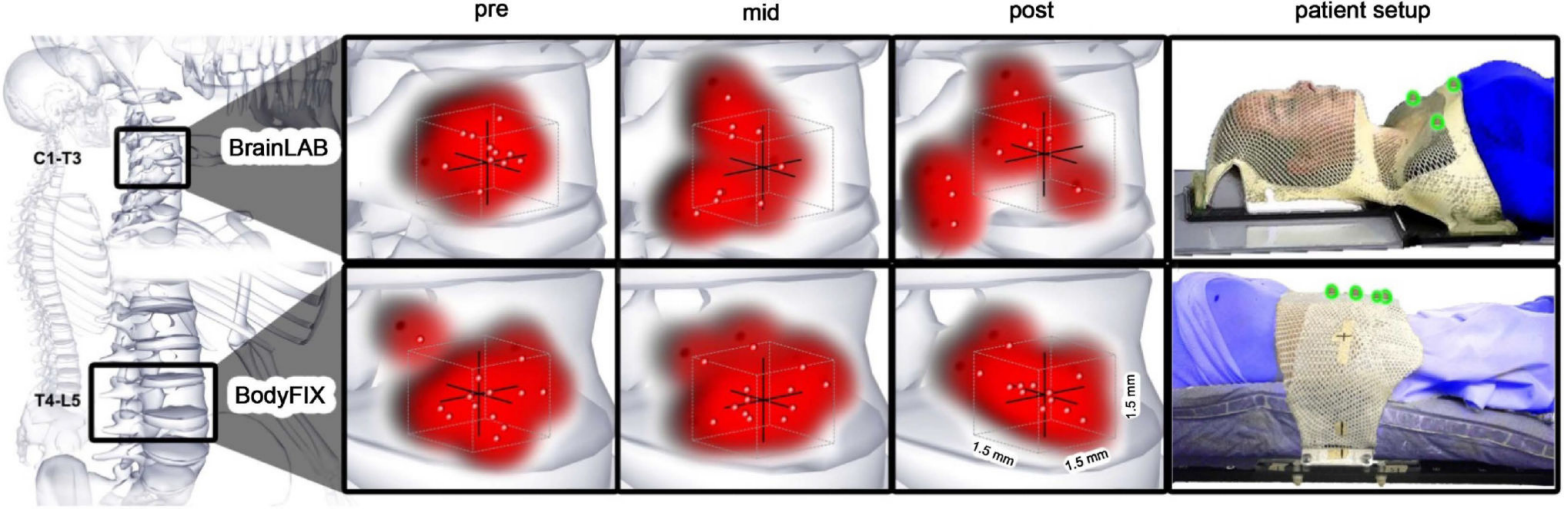
Illustration of patient setup and volumetric projections of residual errors for pre-, mid-, and post-treatments based on immobilization technique. The red points represent where treatment is being delivered within a box, with each side measuring 1.5 mm; the red clouds demonstrate volumetric accuracy and precision of the treatment. There was a significantly greater difference in the average 3D variance of the BodyFIX as compared to the BrainLAB head and shoulder mask immobilization system. The right panel shows patient setup using either approach. The patients are in blue; the immobilization devices are in white; the fiducial markers used are highlighted in green. Illustrations were provided by Nicholas G. Zaorsky, MD.

Based on our translational and rotational coordinates we generated a volumetric projection of residual errors for each treatment fraction based on immobilization technique (Figure 3). We observed a significant difference in volumetric variance of the BrainLAB mask (*p* = 0.001) as compared to the Body FIX immobilization system (*p* = 0.48). There are more data points outside the tolerance residual error box of 1.5 mm in the mid- and post-treatment position verification for the BrainLAB mask immobilization system (upper panel).

## 4. Discussion

In this study we found that the intrafraction motion regardless of immobilization technique was 1.28 ± 0.6 mm using the stereotactic X-ray imaging verification system. This provides acceptable localization accuracy comparable to data obtained from previous spine SBRT studies [15–17]. A comparison of the BodyFIX immobilization system for lower thoracic and lumbar spine to the head and shoulder mask immobilization for cervical and upper thoracic spine showed the following: First, there was significantly greater intrafraction motion in the upper T-C spine as compared to the lower T-L spine in the AP direction: 0.52 ± 0.4 mm and 1.12 ± 0.6 mm, (*p* = 0.02) as well as in 3D: 1.04 ± 0.5 mm and 1.67 ± 0.5 mm, (*p* = 0.04). This suggests that there may be greater C-spine mobility and/or suboptimal mask immobilization. Greater spine mobility is consistent with the anatomy of the C-spine compared to the T-L spine. The laxity of the C-spine allows for maximum mobility in all 6-degrees of freedom. Furthermore, the upper spine is functionally less weight bearing therefore the supporting musculature maybe less developed to withstand strain and stress. As a result, intricate movements in this region of the spine are more common as compared to the more rigid T-spine bound by the thoracic cage and the lumbar spine held in place by a more rigid musculature and the bulk of the abdominal contents. Despite continuous respiratory motion of the ribcage, we did not observe marked movement of the T-spine. This could be due to the fact that the bucket-handle architecture of the rib motion allows for relative motion of the ribs during respiration without affecting the supporting thoracic spine. Alternatively, suboptimal immobilization using the head and shoulder mask immobilization system could explain the increased intrafraction motion in the upper T-C spine. Secondly, the overall treatment time for the upper T-C spine (range 5 to 8 min) was significantly shorter than that of the lower T-L spine (range 5 to 14 min). This suggests that the increased intrafraction motion and residual error for the upper T-C spine treatments is not a result of patient dis-comfort due to increased time on the table. Third, for 4 treatment fractions delivered to the upper T-C spine there was no need for manual correction after mid-treatment verification since residual error was within tolerance. However, the average residual error for this subset was markedly exaggerated upon post-treatment verification (3D: 0.66 mm pre-treatment vs. 0.92 mm mid-treatment vs. 1.96 mm post-treatment).Based on this observation, we strongly recommend that the upper limit of acceptable residual error after mid-treatment verification be made more stringent for the upper T-C spine treatments, since the probability of large intrafractional motion during treatment is very high.

The use of a stereotactic X-ray image verification system offers the advantage of quicker overall treatment time as compared to the kilovoltage-based CT image guidance system (CBCT). The overall treatment time for our patient cohort ranged from 15 – 30 minutes. On the other hand most CBCT based verification systems have longer overall treatment time of 45 min to >60 mins. The increased treatment time is largely due to the time for CBCT image verification rather than “beam-on” duration. Based on the assumption that longer treatment times are often associated with greater positional deviation, a stable position could be hard to maintain in patients with painful lesions necessitating adequate analgesia and comfortable positioning. Thus, a faster image-guided verification system may be more suitable for patients with painful spinal metastases. Furthermore, there is a greater cumulative amount of unnecessary radiation exposure to patients receiving >3 fractions due to the high number of CBCTs delivered during the entire course of treatment. However, CBCT offers an important advantage over ste-reotactic X-ray imaging by providing high-quality volumetric imaging of not only the body structures but also soft tissues including the spinal cord and tumor. This improves positional accuracy and tumor coverage, both of which are critical in the treatment of spine lesions with SBRT. Studies in image-guided radiotherapy for soft tissue-based tumors such as prostate and lung cancer suggest superior positional verification accuracy with CBCT as compared with stereotactic X-ray verification system [18–21]. However, studies comparing these systems for intracranial and spine lesions, as well as head and neck lesions have shown equivalent results [22–26]. The best image guidance system for spine SBRT may be determined by a number of variables including tumor location and characteristics as well as patient-related factors.

Currently, our best immobilization system for treating lesions in the head and neck region is suboptimal. In fact, the problem of poor immobilization in this region has been identified during radiation treatment of head and neck cancers. A great number of set up errors during treatment are often found in the lower neck thus, the need for better immobilization techniques for the lower neck. Several studies on management of cervical spinal fracture have described various C-spine immobilization techniques [27,28]. The best immobilization method is the halo system which requires screws in the base of skull. Although commonly used in radiosurgery for the treatment of intracranial lesions this method may not be ideal for fractionated treatments over the course of multiple days. Other attractive options are the use of the Lerman non-invasive halo system [29] or the cervical-thoracic orthosis (CTO/Minerva brace) [30,31] in combination with the conventional head and shoulder mask for added reinforcement and better immobilization of the lower C-spine. The Lerman halo system has been developed as a non-invasive immobilization system in children. The CTO is designed to provide immobilization of C-and upper T-spine. These provide anterior-posterior, lateral support as well as rotational stabilization. Further research is required to explore alternative C-spine immobilization techniques to improve set up accuracy and decrease intrafraction motion during treatment.

## 5. Conclusion

In this study we show that with our institutional near-rigid immobilization devices and setup based on stereotactic X-ray image guidance, intrafraction motion regardless of immobilization technique was 1.28 ± 0.6 mm. There is greater intrafraction motion for the upper T-C spine BrainLAB mask as compared to the lower T-L spine BodyFIX mask immobilization system. Better immobilization techniques for upper T-C spine SBRT are needed to reduce setup error and intrafraction motion.

## Figures and Tables

**Table 1:** Disease characteristics and treatment schedule for twelve patients treated with stereotactic body radiotherapy to the spine from 2010–2012.

Patient	Disease	Site	Total Dose (Gy)	Fraction number
1	Sarcoma	C1	24	3
2	Meningioma	C5–C6	25	5
3	Renal Cell	C7	18	1
4	Breast	T1–T3	18	3
5	Squamous Cell	T9–T11	18	3
6	Colorectal	T11	15	1
7	Hepatocellular	T11	20	4
8	Breast	T12	16	1
9	Renal Cell	L1	18	1
10	Breast	L3	16	1
11	Schwannoma	L3	20	1
12	Melanoma	L4	20	1

**Table 2:** Translational and rotational residual errors for pre-, mid-, mid-after correction, and post-treatment measurements for upper T-C spine treatments with a tolerance for corrections of 1.5 mm and 2° SD: standard deviation; 3D: three-dimensions; n: number of treatments.

	Translational deviations (mm) mean ± SD						Rotational deviations (degrees) mean ± SD							

Direction	Anterior-Posteror		Superior-Inferior		Right-Left		Pitch		Roll		Yaw		3D variation	
**Upper T-C spine**														
Pre-treatment (n = 12)	0.34	±0.2	0.31	±0.3	0.26	±0.2	0.63	± 0.5	0.68	± 0.3	0.62	±0.4	0.60	±0.2
Mid-treatment														
*Before correction* (n = 12)	0.89	±0.7	0.65	±0.5	0.55	±0.4	0.82	±0.4	0.90	±0.4	0.71	±0.3	1.39	±0.6
*After correction* (n = 8)	0.23	±0.2	0.32	±0.2	0.46	±0.3	0.67	±0.4	0.80	±0.3	0.87	±0.3	0.66	±0.3
Post-treatment (n = 8)	0.65	±0.6	0.55	±0.5	0.61	±0.5	0.63	±0.5	0.75	±0.5	0.67	±0.4	1.22	±0.6
														***p = 0.001***
**Lower T-L spine**														
Pre-treatment (n = 13)	0.41	±0.3	0.40	±0.3	0.54	±0.4	0.40	±0.4	0.38	±0.3	0.77	±0.3	0.89	±0.4
Mid-treatment (n = 13)	0.45	±0.3	0.41	±0.3	0.41	±0.3	0.36	±0.3	0.39	±0.3	0.85	±0.4	0.79	±0.4
Post-treatment (n = 13)	0.44	±0.4	0.31	±0.3	0.35	±0.3	0.46	±0.4	0.43	±0.3	0.84	±0.3	0.71	±0.4
														***p = 0.48***

**Table 3:** Maximum intrafraction variation for all spine, upper T-C spine, and lower T-L spine treatments along each direction and in three-dimensions; n: number of treatments.

	All Spine (n = 25) mm				Upper T-C Spine (n = 12) mm				Lower T-L spine (n = 13) mm			
**Direction**	Mean	Max	Min	SD	Mean	Max	Min	SD	Mean	Max	Min	SD
Anterior-Posterior	0.77	2.26	0.03	±0.6	1.12	2.98	0.23	±0.8	0.52	1.23	0.03	±0.4
Superior-Inferior	0.64	1.57	0.05	±0.4	0.81	1.57	0.36	±0.4	0.51	1.06	0.14	±0.3
Right-Left	0.57	1.33	0.01	±0.4	0.69	1.23	0.21	±0.4	0.59	2.25	0.08	±0.6
3D Variation	1.28	2.77	0.29	±0.6	1.67	3.38	0.79	±0.8	1.04	2.65	0.29	±0.6
